# Nutritional status and behavioral factors of individuals with HTLV-1 and tropical spastic paraparesis (HAM / TSP)

**DOI:** 10.1186/1742-4690-12-S1-P72

**Published:** 2015-08-28

**Authors:** Catia L Carvalho, Tânia FP Mastequin, Rosa Marcusso, Maria RP Gascón, Raquel F Santos, Jorge Casseb, Augusto CP Oliveira

**Affiliations:** 1 Institute of Infectious Diseases Emilio Ribas, Sao Paulo, SP, Brazil

## Introduction

Most of the HTLV-1 carriers remain asymptomatic through life, and genetic and immunological factors may be responsible for the appearance of associated diseases. Objective: To describe the nutritional status and some behavioral factors of individuals with HTLV-1 patients with tropical spastic paraparesis (HAM / TSP).

## Methods

A retrospective, cross-sectional observational study in adults with HAM / TSP patients, both genders attended the Institute of Infectious Diseases Emilio Ribas (IIER) in 2013. The variables studied were age, gender, weight, height, alcoholism, smoking, total cholesterol, glucose, triglycerides, Revised Scale of Motor Osame and Beck Depression Inventory (BDI), Body Mass Index (BMI - WHO, 27).

## Preliminary results

This study evaluated 30 patients, 80% female, mean age 51 years, mean BMI of 24.6, and 40% were overweight and 6.7% obese. With respect to metabolic aspects 6.7% had increased triglycerides, blood glucose and 13.3% presented increased total cholesterol. Importantly, as the behavioral aspects 46.7% had moderate depression and 23.3% needed support of both hands to walk. Regarding the social factors were absent consumption of tobacco and alcohol.

## Conclusion

The assessment of functional capacity changes were identified and commitment gait and balance. Overall, the data showed that patients with moderate depression also had metabolic alterations and overweight. Although it was observed prevalence of normal weight, the excess rate of over weight and metabolic disorders may require monitoring and nutritional interventions. In conclusion, the data suggest that the particular conditions as overweight, metabolic changes impact the daily quality of life of patients with HAM / TSP.

